# Bioaccumulation of Cd in Grapes and Assessment of Human Health Risk

**DOI:** 10.3390/plants15071097

**Published:** 2026-04-02

**Authors:** Ajigul Mamut, Zeyu Wang, Xingwang Ma, Hongbin Liu, Shenghai Pu, Zhaojun Li

**Affiliations:** 1 State Key Laboratory of Oasis Agricultural Environment in Northwest China, Ministry of Agriculture and Rural Affairs, Institute of Agricultural Resources and Environment, Xinjiang Academy of Agricultural Sciences, Urumqi 830091, China; ajigulm@126.com (A.M.);; 2 State Key Laboratory of Efficient Utilization of Arid and Semi-Arid Arable Land in Northern China, Institute of Agricultural Resources and Regional Planning, Chinese Academy of Agricultural Sciences, Beijing 100081, China; 3 Xinjiang Field Scientific Observation and Research Station for Agricultural Non-Point Source Pollution in Korla, Korla 841000, China

**Keywords:** grape cadmium, bio-concentration coefficient (*BCF*), mobility ratio (*MR*), carcinogenic risk (CR)

## Abstract

In the present study, we conducted three treatments with different cadmium concentrations and three different soil types selected for grape cultivation to assess the accumulation and migration characteristics of Cadmium (Cd) in the soil–grape system in different years. The change in Cd fractions in soil and the transfer and accumulation of Cd in different soil–grape systems were analyzed to evaluate the health risks of pulp cadmium accumulation to grape consumers. The results showed that after the planting of the grape, the active Cd fraction increased by 1~3 times and the stable fraction decreased by 1~3 times compared to before planting grapes. It gradually began to stabilize as the cultivation period progressed. The bioaccumulation factor (*BCF*) of Cd in different parts of grape was ranked as: root (0.094~2.590) > stem (0.117~2.112) > leaf (0.008~0.621) > seed (0.010~0.195) > skin (0.000~0.148) > pulp (0.000~0.156). High Cd concentration inhibited the transfer of Cd from the soil and root to the aboveground part of the grape. The Cd of grape pulp has no health risks. Cd pollution significantly altered the soil microbial community, suppressing Actinobacteria while enriching Acidobacteria. The results of this study will help to clarify migration patterns between different soil–grape systems and providing effective data and theoretical support for the management of Cd pollution in vineyard soils.

## 1. Introduction

Heavy metals that accumulate in the soil are absorbed into the fruit via plant roots, leading to problems with food quality and safety issues. The accumulation of heavy metals from soil into plants mainly depends on the uptake mechanisms, the physicochemical properties of the soil and the chemical speciation of metals and metalloid(s) in the soil [1]. Cadmium (Cd) is usually found in the Earth’s crust, released by human activities or natural factors, such as zinc–lead and copper, or enters the water by leaching, or is released into the air via forest fires or volcanic eruptions. In 2018, China accounted for 32% of the global Cd production, making it the world’s largest Cd producer and the world’s largest Cd emitter [2]. The area of heavy metal contaminated soil caused by human activities in China is growing at a rate of 4.26106 hectares per year, a significant proportion of which is Cd contaminated soil [3]. Moreover, Cd is migratory and long-term, and the Cd in soil is transferred to plants and accumulated, have a hidden danger to food safety. As can be seen, the current situation of Cd pollution in China is not optimistic and should be taken seriously to prevent further deterioration.

The migration of heavy metals in soil–plant systems has also been investigated in many previous studies. As staple crops, wheat and rice can easily accumulate heavy metals in their grains [4,5,6]. Therefore, the quantitative relationship of soil heavy metals in the soil–grape system, the law of enrichment and transfer, and the change in soil heavy metal forms over time are the prerequisites for preventing and remedying the heavy metal pollution in vineyard soil and ensuring the sustainable development of grape industry. In addition, while previous reports have also evaluated heavy metals in fruits (pear, mango, banana, jackfruit and orange) [7], vegetables [8,9], rice [10], wheat [11], and edible mushrooms [12] and the associated health risks, few researchers have addressed the migration of heavy metals in soil–grape systems [13,14,15]. But no one has studied the accumulation of exogenous Cd in different soil types of soil in grapes, the migration between grapes and soil, the transformation rule and systematic evaluation of the health risk of Cd in grapes grown in different soils under exogenous Cd stress. In general, vineyard soils are easily degradable and more susceptible to contamination. In this context, heavy metal pollution of vineyard soils is a major environmental problem that can affect crop productivity, food quality and human health [16]. Since table grapes are one of the fruits that people eat daily, it is of great practical importance to assess the health risk from heavy metals caused by table grapes.

The transformation of heavy metal forms has a direct effect on its migration and enrichment capacity in soil–plant system. Therefore, studying the morphological changes in Cd in soil before and after grape planting is the basic requirement for studying Cd migration in the soil–grape system. In this study, we analyzed the changes in soil Cd content and Cd morphology in aquic soil, brown soil and red soil, with the same fruit of three treatments in the second and third year of potting (2022–2023), and the corresponding grape samples (roots, stems, leaves, pulp, skins and seeds), and compared and analyzed the changes under different cultivation years, different exogenous Cd treatments and different soil types. Changes in Cd form before and after grape planting, enrichment characteristics of Cd in different parts of grapes and their influencing factors, transfer and migration characteristics of exogenous soil Cd in the soil–grape system, health risks of Cd in grape pulp to consumers, etc., were examined. The aim is to provide a scientific basis for the prevention and control of heavy metals in grape-growing areas, the formulation of safety thresholds for Cd in vineyard soil and the protection of grape consumers’ health level. The results of this study will provide sufficient data and theoretical support and guidance for grape production management and environmental protection.

## 2. Materials and Methods

### 2.1. Experimental Design

**Figure 1:**
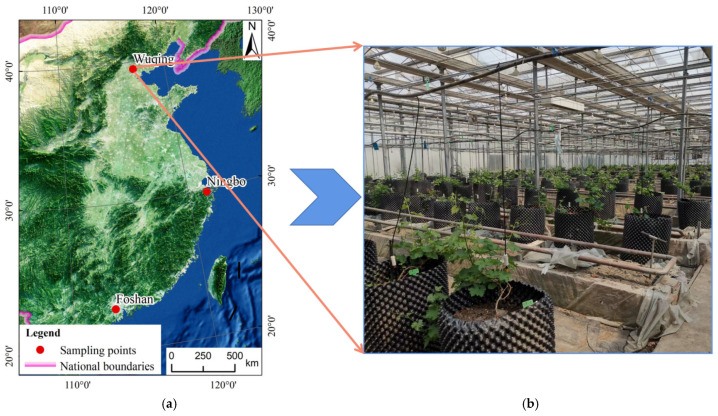
Sampling sites (**a**) and cultivation site (**b**).

A three-year pot experiment (2021–2023) was conducted, with sampling time from August 2022 to August 2023, in a greenhouse at the Innovation Base of the Academy of Agricultural Sciences in Tianjin, China (Figure 1, Table S1) to investigate the transfer and accumulation of different Cd concentrations in different types of soil–grape systems and the associated health risks to grape consumers. Three different types of vineyard soil (fluvo-aquic soil, derived from the Tianjin Academy of Agricultural Sciences agricultural innovation base grape vineyard; brown soil, derived from the China Ningbo Jiangbei Adong fruit professional cooperative vineyard; and red soil from the China Foshan Agricultural Science Institute vineyard; (Figure 1a, Table 1). The experiment included three treatments: CK—without Cd addition; Cd_l_—low Cd, i.e., addition of Cd at 1 time the national standard [17]; Cd_h_—high Cd, i.e., addition of Cd at 2.5 times the national standard [17]; see details in Table 2. Cadmium chloride stock solution (CdCl_2_ 2.5H_2_O) was added to air-dried soils to produce different Cd concentrations, and each treatment had three replicates. At the end of the 90 days of aging, the grape seedlings were transplanted in July 2021. The grape seedlings (Muscat Hamburg; *Vitis vinifera* L.) were raised in unpolluted soil at the Grape Research Institute of the Innovation Base of Tianjin Academy of Agricultural Sciences. Grape seedlings of a similar size were selected and planted into the soil. The cultivation and management of the grape seedlings were identical. The cultivation experiment was conducted in the glass shed of the Grape Research Institute of the Innovation Base of Tianjin Academy of Agricultural Sciences (Figure 1b).

#### 2.1.1. Soil Samples

Twenty-seven pairs of topsoil (0–20 cm) and corresponding grape samples (root, stem, leaf, and fruit) were collected during the harvest seasons of 2022 and 2023. Soil pH and organic matter (SOM) were measured with a pH meter (soil:water = 1:2.5) and the K_2_Cr_2_O_7_ oxidation method, respectively. The chemical forms of Cd in soils were sequentially extracted using a modified BCR procedure [18], which partitions heavy metals into acid-soluble (F1), reducible (F2), oxidizable (F3), and residual (F4) fractions (Table S2). This method is widely recognized for its stability and precision in assessing metal mobility [19,20]. In the standard soil sample (GBW07406-GSS-6) added with Cd, the recovery rate of Cd was 103% ± 3%. In addition, when the four fractions of Cd were determined by the BCR sequential extraction procedure, the total Cd content was simultaneously measured via the aqua-regia digestion method (tri-acid digestion method). The difference between the sum of the four fractions and the total Cd content was negligible. The recovery was 96% ± 2%, which verified the reliability of the BCR sequential extraction procedure. Furthermore, to ensure the absence of contamination, the Cd content in the soil samples was determined prior to exogenous Cd.

Simultaneously, fresh soil samples were collected for microbial analysis. The V3–V4 region of the bacterial 16S rRNA gene was amplified with specific primers [21,22] and subjected to high-throughput sequencing by Shanghai Meiji Bio-Technology Co., Ltd. (Shanghai, China). Detailed PCR procedures are provided in Supplementary Tables S4–S6.

#### 2.1.2. Grape Samples

The samples of grape roots, stems, leaves and fruits were first washed with tap water to remove adhering soil particles and then rinsed with deionized water. Fruit samples were separated into seeds, pulp and skin. All separated samples, as well as the root, stem, and leaf samples, were dried in an oven at 70 °C and then pulverized in an agate mortar and mixed with stainless steel balls. The pretreated samples were then stored in sealed polyethylene bags at 4 °C for further analysis. All samples (0.2~0.5 g) were digested in a microwave digester (ETHOS 1, Advanced Microwave Digestion System, Milestone, Milan, Italy) [23] for 45 min (Table S3).

### 2.2. Data Analyses

#### 2.2.1. Bioaccumulation Coefficient (BCF)

Bioaccumulation factor (*BCF*) is an index of the ability of plants to accumulate heavy metals from soil. The higher the BCF of a plant for a heavy metal, the easier the plant can absorb the heavy metal from the soil and the greater its absorption capacity. If the *BCF* is ≥1, it indicates that the plant is highly enriched in the heavy metal. The *BCF* was calculated using the following formula:

(1) $$BCF=\frac{C_{grape}}{C_{soil}}$$

where C_grape_ represents the heavy metal content in grape different part (mg/kg, dry weight) and C_soil_ represents the total heavy metal content in the corresponding soil sample (mg/kg) [24].

#### 2.2.2. Transfer Factor (TF)

The transfer factor (*TF*) reflects the ability of the Cd absorbed by the root system to migrate to aboveground plant parts or other organs. The higher the transfer coefficient of a heavy metal in a plant, the more cadmium is absorbed by the roots and transported above ground, and presumably, the greater the likelihood of subsequent transport to the fruit, which is more susceptible to cadmium contamination. The calculations were based on the following formula:

(2) $$TF=\frac{C_{abovegroundpart}}{C_{root}}$$

#### 2.2.3. Mobility Ratio (MR)

The mobility ratio (MR) of heavy metals is an important measure of the ability of heavy metals to transfer from the soil to the aboveground parts or various organs of the plant. The greater the mobility of heavy metals in a particular soil–plant system, the more likely the plant is to take up the heavy metal from the soil, and the greater its uptake capacity [25]. For cadmium (Cd) specifically, its mobility across different soil types is primarily regulated by key soil physicochemical properties that mediate Cd speciation and bioavailability. In calcareous soils (pH 7.5–9.0), Cd tends to form low-mobility carbonate-bound or hydroxide precipitates, which limits its transfer to plants; however, high salinity in such soils (e.g., elevated Na^+^ and Cl^−^ concentrations) can enhance Cd mobility by forming soluble Cd-chloride complexes (e.g., CdCl^+^, CdCl_2_^0^) that are easily absorbed by plant roots [26,27]. In acidic soils (pH < 6.0), the low pH reduces Cd adsorption onto soil colloids (e.g., clay minerals, organic matter), increasing the proportion of exchangeable Cd (the most mobile fraction), thereby promoting Cd migration to aboveground plant parts [28,29]. Additionally, soil organic matter (SOM) content exerts a dual effect: low SOM levels reduce Cd complexation, enhancing mobility, while high SOM stabilizes Cd through chelation with carboxyl/hydroxyl functional groups, decreasing its mobility [30,31]. The MR was calculated using the following formula:

(3) $$MR=\frac{C_{abovegroundpart}}{C_{soil}}$$

#### 2.2.4. Health Risk Assessment

Health risk assessment evaluates the potential health effects of doses of a pollutant delivered to humans in any form [32]. The values most commonly used in soil studies are from the US EPA guidance for human health risk assessment [28,30,33,34]. Health risk assessments first appeared in medical research in the 1930s, and heavy metal contamination became prevalent. Since Cd is identified as a carcinogenic heavy metal, we used the carcinogenic risk index (*CRI*) to estimate the lifetime probability of grape consumers developing cancer due to the consumption of Cd-contaminated grapes. Carcinogenic risk index (*CRI*) has been used to estimate the probability that a person will develop cancer due to exposure to a carcinogenic or potentially carcinogenic substance during his or her lifetime [4,35]. The calculations were made based on the following formula:

(4) $$EDI=\frac{C\times IR\times EF\times ED}{BW\times AT}$$

(5) CRI=EDI×SF

where *EDI* (mg/kg. BW/day) is the estimated daily intake of the considered metal via grape consumption; *C* is the concentration of the considered heavy metals in grape pulp (mg/kg, fresh weight); *IR* is the ingestion rate of grapes (adults: 0.1768 kg/d; children: 0.0681 kg/d) [13]; *EF* is the exposure frequency (d/a), 90 days in a year; *ED* is the exposure duration (a), 70 years for an adult’s life and 12 years for a child’s life; *BW* is average body weight (adults: 60 kg; children: 25 kg); and *AT* is the average exposure time (d), calculated by 365 days/year × *ED*. *SF* is the slope factor of the carcinogenic or potentially carcinogenic substance. The *SF* for Cd is 0.0085 (mg/kg/day)^−1^ [32]. A *CRI* value of less than 10^−4^ was considered acceptable for grape consumers [4]. Exceeding this threshold increases the risk of developing several cancers, with the most well-documented being lung cancer, followed by prostate cancer, kidney cancer, and bladder cancer.

#### 2.2.5. Analysis of Microbial Community Data

After sequencing, raw reads were quality-filtered to remove low-quality sequences (Q-score < 20) and chimeric sequences using the DADA2 plugin in QIIME 2 (Version 2022.11). High-quality sequences were clustered into Operational Taxonomic Units (OTUs) at a 97% sequence similarity threshold. Microbial diversity indices were calculated based on the OTU table:

α-diversity indices: Shannon index, Simpson index, Chao1 index, and ACE index were computed using the vegan package in R software (Version 4.3.1) to evaluate the richness and evenness of bacterial communities within individual samples;

β-diversity indices: Bray–Curtis distance and Unweighted Unifrac distance were calculated to assess the dissimilarity of bacterial communities among different samples. Non-metric Multidimensional Scaling (NMDS) was used to visualize the β-diversity patterns.

#### 2.2.6. Statistical Analysis

Excel and Origin Pro 2022b were used for data analysis. Bidirectional analysis of variance (ANOVA) and Pearson correlation coefficient were used to determine the relationship between total Cd (after treatment), Cd morphology, pH, SOM and CEC in soil and grape Cd. All observed data followed a normal distribution (Shapiro–Wilk test, *p* > 0.05) and met the requirement of homogeneity of variance (Levene’s test, *p* > 0.05).

## 3. Results

### 3.1. Changes in Cd Morphology in Vineyard Soil in Different Years

**Figure 2:**
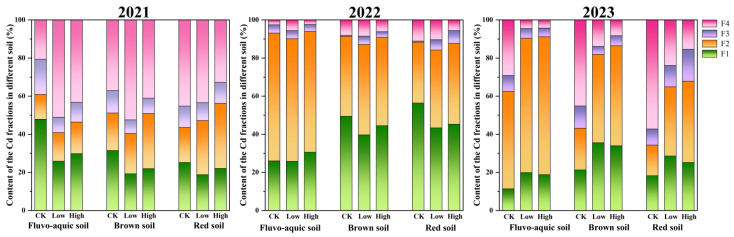
Changes in Cd morphological content in vineyard soil before and after grape planting. (F1: acid soluble fraction; F2: reducible fraction; F3: oxidizable fraction; F4: residual fraction).

The chemical speciation of heavy metals in soil is a critical indicator of their mobility and bioavailability. Changes in Cd speciation in three soil types over three years are shown in Figure 2. Before planting (Figure 2, 2021), residual fraction (F4) was the dominant form in most soils (33–53%), while the oxidizable fraction (F3) was the least abundant (7–18%). After two years of grape cultivation (Figure 2, 2022), the proportions of the acid-soluble (F1) and reducible (F2) fractions increased markedly, collectively accounting for 84–94% of total Cd, indicating a significant activation of Cd. Compared to pre-planting, F1 and F2 increased by 1–31% and 8–34%, respectively, whereas F3 and F4 decreased by 3–14% and 17–45%.

In the third year (Figure 2, 2023), a reversal of this trend was observed, with F3 and F4 proportions increasing while F1 and F2 decreased, suggesting a gradual transition of active Cd toward more stable forms. This stabilization was most evident in the control groups. In contrast, soils treated with higher exogenous Cd levels showed slower stabilization, as reflected by their lower F4 proportions compared to the low-Cd treatment groups.

### 3.2. Enrichment Characteristics of Cd in Different Parts of Grape (BCF)

**Figure 3:**
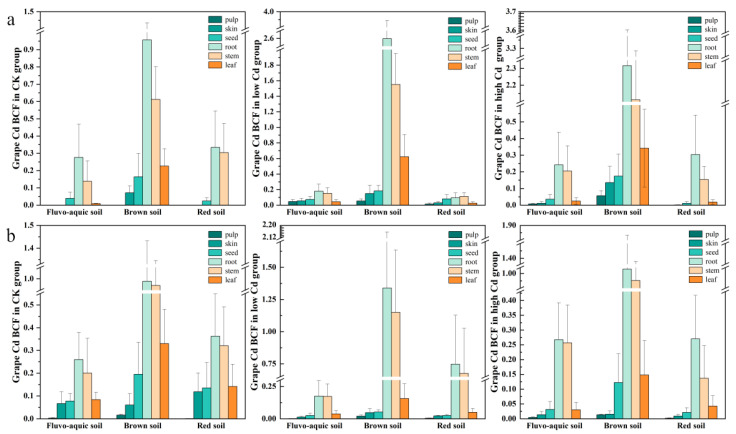
Bioaccumulation factor (BCF) of cadmium (Cd) in different organs of grapes grown in three soil types under CK, low, and high exogenous Cd treatments (2022 vs. 2023). Note: (**a**): data collected in 2022; (**b**): data collected in 2023. Treatments: CK (control group, no exogenous Cd added), how (low exogenous Cd treatment), high (high exogenous Cd treatment).

The migration and transformation of cadmium (Cd) in the soil–plant system are influenced by multiple factors, including crop type and soil properties. The bioaccumulation factor (BCF) effectively reflects the enrichment capacity of heavy metals in various plant parts [33]. As shown in Figure 3, Cd enrichment varied significantly across soil types, exogenous Cd levels, and grape tissues. Grape plants in more acidic brown soil (pH ≈ 5.73) exhibited significantly higher Cd enrichment than those in fluvo-aquic and red soils, consistent with the principle that low pH enhances metal desorption and mobility [30].

In the second year, the overall Cd enrichment in grapes followed the order of low Cd > high Cd > CK in fluvo-aquic and brown soils, while the order was CK > high Cd > low Cd in red soil. Across all soils, Cd was predominantly accumulated in roots and stems, with the distribution generally following the order of root > stem > leaf/seed > skin > pulp. Notably, the BCF values for roots and stems in brown soil under Cd treatments exceeded 1, indicating significant accumulation, whereas values in other soils remained below 1.

By the third year, the overall Cd BCF in grapes decreased compared to the second year, though the tissue-specific order (root > stem > leaf > seed > skin > pulp) remained consistent. Soils and treatments exhibited distinct temporal trends: for instance, roots and stems in the low-Cd group showed increased BCF, while seeds and skins generally decreased. The final BCF patterns ranked as low-Cd > high-Cd > CK in brown and red soils, but as CK > high-Cd > low-Cd in fluvo-aquic soil, highlighting the persistent influence of both soil properties and exogenous Cd concentration on long-term metal accumulation.

### 3.3. Transfer and Migration Ratio Characteristics of Cd in Soil–Grape System

**Figure 4:**
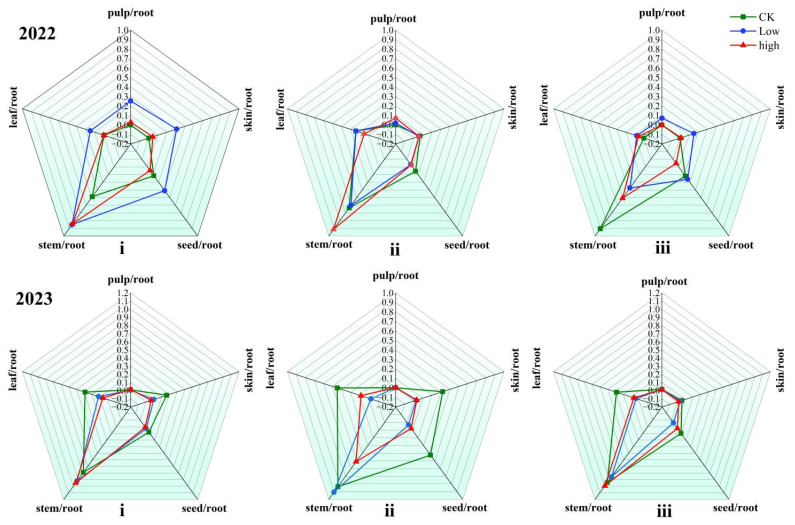
Cadmium (Cd) transfer ratio between grape roots and aboveground organs across different soil types (2022 vs. 2023). Note: Temporal groups: top row = 2022; bottom row = 2023. Soil types (Roman numerals): i = fluvo-aquic soil; ii = brown soil; iii = red soil. Treatments: CK (control group, no exogenous Cd added; green line), low (low exogenous Cd treatment; blue line), high (high exogenous Cd treatment; red line). Axes: each axis represents the Cd transfer ratio of one aboveground organ relative to roots (pulp/root, skin/root, seed/root, stem/root, leaf/root).

Following uptake by grape roots, cadmium (Cd) is translocated to various aerial tissues via the transpiration stream. The transfer factor (TF) of Cd from roots to different organs varied significantly depending on soil type, exogenous Cd concentration, and cultivation year (Figure 4). Across all conditions, the root-to-stem TF was consistently the highest, indicating efficient Cd transport into the stem after root absorption, a finding supported by other studies [28]. In contrast, the root-to-pulp TF was the lowest, demonstrating a strong restriction on Cd transfer into the edible fruit portion. In the second year, low Cd treatment generally promoted Cd translocation, particularly in fluvo-aquic and red soils, where TFs in most low-Cd groups exceeded those in the control and high-Cd groups. However, this pattern shifted in the third year, where high Cd concentration notably inhibited Cd transfer from roots to aerial parts. In most treatment groups, TFs in the third year were lower than in the control, suggesting that prolonged high Cd exposure impaired the plant’s internal translocation capacity.

Detailed interannual comparisons revealed distinct TF trends among soil types and treatments. For instance, in the third-year control groups, TFs to pulp, skin, and seed often increased compared to the second year. In contrast, most low- and high-Cd-treated groups showed declines in TFs to leaves, skins, and seeds. These results collectively demonstrate that Cd transfer within grapes is a dynamic process, modulated by the interplay of soil properties, metal exposure level, and plant growth duration.

Depending on the soil type, exogenous Cd concentration and years of cultivation, the migration ratio (*MR*) between the grape soil and the aboveground part of the grape also varied (Table 3). The *MR* between soil and aboveground parts of the grapes in brown soil under different treatments and in the different years of cultivation was higher than in the fluvo-aquic and red soils and increased with the increase in exogenous Cd in the second year, while the *MR* of the three treatments in the third year was lower than that of the corresponding treatments in the second year. The average *MR* of acidic soil (brown soil) is about 3 to 20 times that of alkaline soil (red soil), indicating that Cd has high bioavailability in acidic soils and is easily transferred from soil to grape. *MR* is negatively correlated with soil pH, which is consistent with previous research findings [34].

### 3.4. Evaluation of Grape Cd’s Health Risk to Consumers

**Figure 5:**
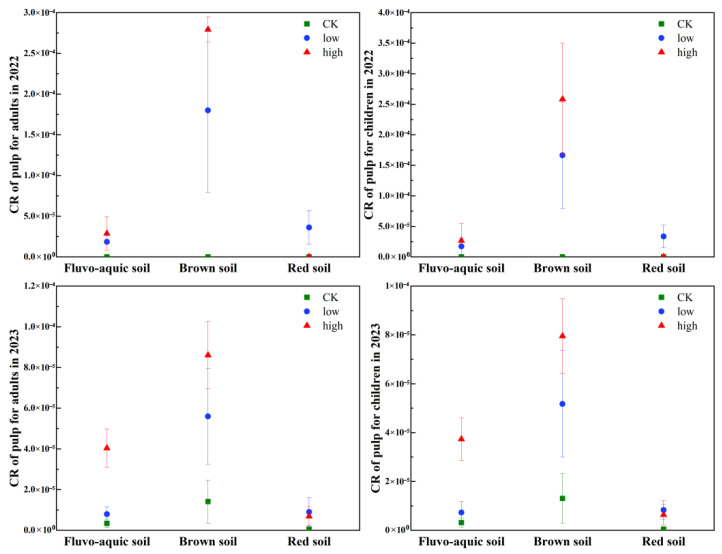
Carcinogenic risk (CR) of grape pulp for adult and child consumers across different soil types and exogenous Cd treatments (2022 vs. 2023). Note: CR definition: Carcinogenic risk, reflecting the potential cancer risk caused by long-term consumption of grape pulp. Soil types: fluvo-aquic soil, brown soil, red soil. Treatments: CK (control group, no exogenous Cd added; green), low (low exogenous Cd treatment; blue), high (high exogenous Cd treatment; red). Population groups: top row = 2022 data; bottom row = 2023 data; left column = adults; right column = children. Safety threshold: The dashed line in the figure represents the acceptable carcinogenic risk threshold (1 × 10^−6^) recommended by the U.S. Environmental Protection Agency (USEPA). Values above this line indicate non-negligible cancer risk.

The health risk of Cd in grape pulp, evaluated by the carcinogenic risk (CR) index, varied with soil type, Cd treatment, and cultivation year (Figure 5). The CRI for consumers was consistently higher in acidic brown soil than in fluvo-aquic and red soils. In the second year, the CRI values for both adults and children in brown soil under low and high Cd treatments exceeded the 1 × 10^−4^ threshold, indicating a potential health risk. Although the CRI in brown soil decreased in the third year, it remained at an acceptable medium risk level (10^−5^ < CRI ≤ 10^−4^), confirming that acidic conditions enhance Cd bioavailability and associated consumer risk. These findings align with the observed Cd speciation, plant enrichment, and translocation patterns (Figure 2 and Figure 3, Table 3).

Temporal trends revealed that health risks generally decreased from the second to the third year in most treatment groups, particularly in brown soil. However, an inverse trend was observed in the CK and high-Cd groups of fluvo-aquic soil and red soil, where CRI increased over time. Overall, the risk for children was consistently lower than for adults. The risk ranking across soils was consistently brown soil > fluvo-aquic soil > red soil, with red soil pulp posing negligible risk (CRI < 10^−6^) in the third year. Given that grapes are a widely consumed fruit, and Cd is a highly toxic element known to accumulate in agricultural soils through practices like phosphate fertilizer application [35], the Cd content in the edible pulp warrants significant attention. Although the assessed risks were within acceptable limits in most scenarios, the persistent medium risk in acidic soils underscores the need for proactive environmental management in grape production areas to safeguard long-term food safety and public health.

### 3.5. Effects of Cd Stress on Soil Microorganisms in Vineyards

The presence of heavy metals often severely affects microbial growth as heavy metals alter the permeability of cell membranes and disrupt other physical and chemical properties within microbial cell [36]. In this study, three exogenous Cd treatments (control: CK; low-Cd: L; high-Cd: H) stress were investigated. Soil samples were collected from three different soil types: fluvo-aquic soil (F), brown soil (B), and red soil (R). The soil samples were then subjected to 16S RNA gene amplicon sequencing to assess the impact of the Cd treatments on microbial communities in the different soil types.

The composition of the soil microbial community varied according to soil type, concentration of exogenous Cd and cultivation age. The top six dominant bacterial groups in the bacterial community of the vineyard soil are Actinomyces, Proteobacteria, Chloromyces, Acidobacteria, Firmicutes and Gemmatimonadetes. In 2022, a total of 1,074,832 high-quality sequences were obtained from the soil bacterial community analysis of vineyards. The high-quality sequence length was mainly distributed in 416 bp, which was divided into 1 phylum, 40 classes, 130 orders, 312 families, 498 genera and 940 species. A total of 2,198,612 high-quality sequences were obtained from the analysis of soil bacterial community in 2023. The high-quality sequence length was mainly distributed in 415 bp, which was divided into 1 phylum, 45 classes, 149 orders, 378 families, 635 genera and 1315 species.

#### 3.5.1. Structural Characteristics of Bacteria at Phylum Level Under Cd Stress

**Figure 6:**
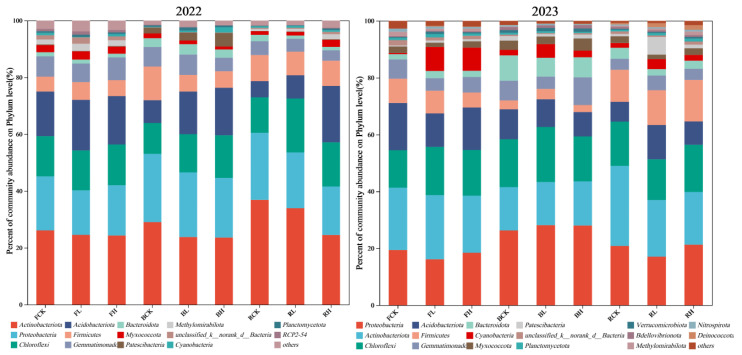
Changes in bacterial community composition (phylum level) in grape rhizosphere soil across different soil types and exogenous Cd treatments (2022 vs. 2023). Note: FCK: control group in fluvo-aquic soil; FL: low-Cd treatment group in fluvo-aquic soil; FH: high-Cd treatment group in fluvo-aquic soil; BCK: control group in brown soil; BL: low-Cd treatment group in brown soil; BH: high-Cd treatment group in brown soil; RCK: control group in red soil: RL: low-Cd treatment group in red soil; RH: high-Cd treatment group in red soil.

According to the results of classification annotation at the structural level (Figure 6), a total of six categories of bacteria with a high relative abundance of more than 5% were identified in the soil samples of the rhizosphere of grapes treated with exogenous Cd in 2022, in the following order: Actinobacteria (27.44%), Proteobacteria (20.15%), Chloroflexi (14.34%) and Acidobacteria (13.78%), Firmicutes (7.42%), and Gemmatimonadetes (5.97%). There are six bacteria with relative abundance of more than 5% in 2023, the same species as in 2022, but the order of abundance is different: Proteobacteria (21.73%), Actinobacteria (19.68%), Chloroflexi (16.07%), Acidobacteria (11.05%), Gemmatimonadetes (8.11%), Firmicutes (7.68%). From the above data, it can be seen that the abundance of each predominant bacteria had certain differences in the different cultivation ages, soils and treatments.

In this experiment, the relative abundance of Proteobacteria in the three soils ranked second in the second year of cultivation, but in the third year, the relative abundance of Proteobacteria in the three soils was the highest, which is consistent with previous research results [37,38]. Proteobacteria have attracted much attention due to their excellent ability to decompose complex organic matter and high resistance to numerous heavy metal contaminants. Recent studies have shown that several heavy metal oxidase genes carried by Proteobacteria play a crucial role in their heavy metal tolerance [39]. These genes are involved in the transformation process of heavy metals, effectively reduce the bioavailability of these harmful elements; this allows Proteobacteria to survive and function in environments containing heavy metals [39]. Chloroflexi is the third bacterial phylum that is relatively abundant in the soil of this study. Recent studies have shown that the abundance of Chloroflexi in soil increased with the addition of Cd, suggesting that these microorganisms may be better adapted to environments with high Cd concentration [40,41].

Other studies have shown that under the condition of heavy metal pollution, the activity and quantity of Acidobacteria increased significantly, which may be related to their unique physiological mechanism to cope with and transform heavy metals [42]. In this study, in the second year of cultivation, the relative abundance of Acidobacteria in each soil increased with the increase in exogenous Cd. However, in the third year, the relative abundance of Acidobacteria decreased to varying degrees compared to the second year, which may be related to the changes in Cd form and other factors in grape rhizosphere soil. Previous studies have shown that Proteobacteria continue to dominate in sediments, surface water and soil heavily polluted with heavy metals [43,44]. Proteobacteria are generally not affected by heavy metals in soil and have a relatively fast growth rate under conditions of Cd pollution [45,46].

At the genus level, the Chao index (community richness) and Shannon index (community diversity) were selected in this study to evaluate the α-diversity of soil microorganisms in different types of vineyards under different exogenous Cd treatments (Table S6). The Shannon and Chao indices are higher in 2023 than in 2022 for all soils and treatments. The results show that the cultivation time had significant influence on bacterial abundance in the soil, but only minor influence on bacterial diversity.

#### 3.5.2. Effect of Cd Contamination on Bacterial Community Composition in Soil

**Figure 7:**
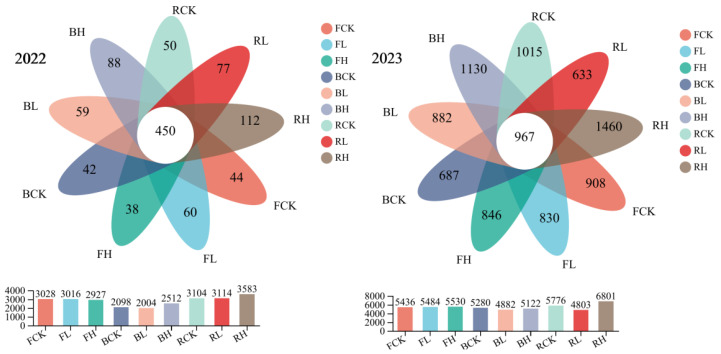
Venn diagram of bacterial communities treated with Cd at different years (2022 and 2023) and concentrations. Note: FCK: control group in fluvo-aquic soil; FL: low-Cd treatment group in fluvo-aquic soil; FH: high-Cd treatment group in fluvo-aquic soil; BCK: control group in brown soil; BL: low-Cd treatment group in brown soil; BH: high-Cd treatment group in brown soil; RCK: control group in red soil; RL: low-Cd treatment group in red soil; RH: high-Cd treatment group in red soil.

Figure 7 shows the composition of bacterial communities in different soils and at different Cd concentrations. According to the 97% sequence similarity criterion, 919 OTUs were identified by the 2022 consensus, of which 450 OTUs were common to all samples. In the two red soil samples treated with high Cd, the number of unique OTUs reached 112, which was higher than the other soils. By 2023, the consensus identified 9358 OTUs, of which 967 were common to all samples. In the red soil samples treated with high Cd, the number of unique OTUs was 1460, which was also higher than other soils. The OTU values of all soils and all treatments in 2023 were higher than those of the same soil and treatment in 2022. In addition, as can be seen from the bar chart below (Figure 7), the total number of bacteria in red soil treated with high Cd concentration is the highest in two years, being 3583 and 6801 in 2022 and 2023, respectively. The total number of bacteria in each soil and treatment was higher in 2023 than in 2022. The main reason was that in the third year, the form of Cd in the vineyard soil began to stabilize and the content of available Cd gradually decreased, so that the toxic effect of Cd in the soil on microorganisms was gradually weakened. Another reason is that under the stress of heavy metal pollution with different degrees, all tested bacteria can transform or immobilize cadmium (Cd) in the soil by producing specific metabolites (e.g., organic acids, chelating agents) or activating physiological regulation mechanisms (e.g., cell membrane adsorption, intracellular accumulation), thereby enhancing their tolerance to heavy metals [42,47].

#### 3.5.3. Relationship Between Soil Bacteria and Environmental Factors in Vineyard

**Figure 8:**
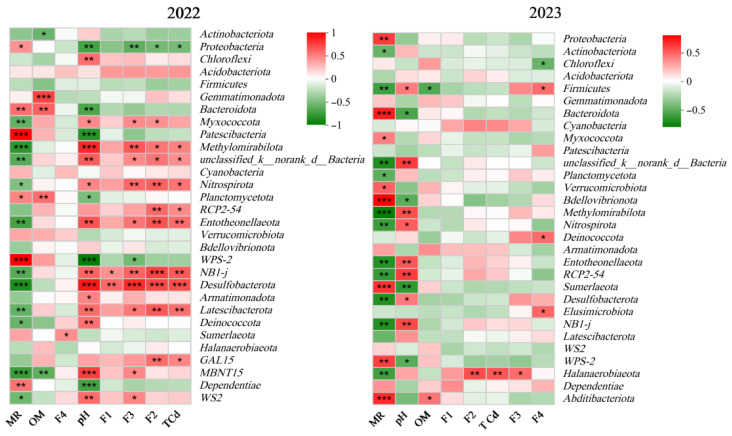
Correlation between rhizosphere soil bacterial community and environmental factors. (Note: * means *p* ≤ 0.05, ** means *p* ≤ 0.01, *** means *p* ≤ 0.001; F1: acid soluble state Cd; F2: oxidation-bound state Cd; F3: organic binding state Cd; F4: residual state Cd; TCd: total cadmium; MR is the migration coefficient of Cd between soil and grape).

To better understand the correlation between the rhizosphere soil bacterial community (TOP 30) and environmental factors (including soil pH, OM, total Cd and its morphological content) and the soil–grape mobility (MR) of Cd, a correlation analysis was performed with the data from the second and third years of cultivation (Figure 8). The correlation heat map shows that the microorganisms in the 2023 soil samples are less sensitive to environmental factors. In particular, the total content of F1, F2, F3 and Cd rapidly weakened the influence of soil microorganisms. Desulfobacterota, BH1-J, Entotheonellaeota, Nitrospira and Methyspora were all significantly negatively correlated with MR in the second and third years of cultivation. These bacteria inhibited the migration of Cd between soil and grape. WS2, Bacteroides, Proteobacteria and other bacteria were significantly positively correlated with MR. These bacteria promote Cd migration between soil and grapes.

Desulphurobacteria are beneficial microorganisms, suggesting that their microbial environment in the soil rhizosphere is to some extent more favorable for forest growth [48]. The correlation of Desulfobacterota with MR decreased from −0.724 (*p* ≤ 0.001) in 2022 to −0.572 (*p* ≤ 0.01) in 2023; *Entotheonellaeota* was negatively correlated with MR, and the two-year correlations were −0.593 (*p* ≤ 0.01) and −0.564 (*p* ≤ 0.01), respectively. The maximum adsorption capacities of Pb, Cu and Cd by *Enterobacter* were 50.9, 32.5 and 46.2 mg/g, respectively [49]. The results showed that *Entotheonellaeota* inhibited Cd migration between soil and grapes through its ability to adsorb and fix Cd. Soil pH and organic matter have been widely reported to have important effects on soil bacterial communities [50,51,52]. Some scholars have found that low pH increases the availability of heavy metals, so low pH and low organic matter are very unfavorable for the living environment of microorganisms [53,54]. However, the response of microorganisms to heavy metals is not uniform; some of them are very sensitive to heavy metals and other pollutants, while others are less sensitive [55]. *RCP2-54* is a low-abundance rare taxon with no complete species annotation in public databases, and it is only classified as unclassified bacteria. In this study, it was significantly and positively correlated with total cadmium (TCd) and the oxidation-bound state (F2) in 2022. In addition, the results from 2023 showed that RCP2-54 was significantly positively correlated with pH and significantly negatively correlated with MR. At present, there are no specific studies on the association between *RCP2-54* and heavy metals. This result warrants further investigation. Therefore, the correlation between microorganisms in the grapevine rhizosphere and various environmental factors and MR Varies according to the cultivation age.

## 4. Discussion

### 4.1. Temporal Dynamics of Cd Bioavailability

The chemical form of cadmium (Cd) in soil governs its mobility, bioavailability, and toxicity. These forms are dynamic and can interconvert with changing environmental conditions. Commonly identified using the modified BCR sequential extraction procedure, Cd is partitioned into four fractions: weak acid soluble (F1), reducible (F2), oxidizable (F3), and residual (F4). The residual fraction (F4) is stable and poses minimal environmental risk, while the bioavailability generally decreases in the order of F1 > F2 > F3 > F4 [56]. Plant roots play a crucial role in activating Cd through the release of secretions like organic acids, which enhance the mobilization of Cd and facilitate the transformation of its stable forms into more bioavailable ones [57,58,59,60]. This study confirms that roots and their secretions promote Cd activation, consistent with other findings [61]. The transformation of Cd forms is influenced by the activation level in the rhizosphere and plant uptake of exchangeable Cd [62]. Furthermore, exogenous factors, particularly the concentration of added Cd, significantly impact morphological transformation and stability. This study demonstrates that higher concentrations of exogenous Cd require a longer time to reach equilibrium, aligning with our previous research [63,64].

### 4.2. Compartmentalization of Cd in Grape Tissues

Cd enrichment in grape roots significantly exceeded that in other plant parts across different cultivation years, soils, and treatments, consistent with previous studies [65,66,67,68]. This is attributed to the abundance of exchange sites in root cell walls that immobilize heavy metal ions, limiting their translocation to aerial tissues [69]. In soil–grape systems, Cd accumulation varied markedly with soil type, following the sequence: brown soil (1.786 mg/kg) > fluvo-aquic soil (0.364 mg/kg) > red soil (0.213 mg/kg). Soil pH plays a critical role, as lower pH increases metal desorption, while pH > 7 promotes more stable metal forms [70]. The Cd content in grape organs decreased progressively from roots to fruits, with approximately 85–90% of Cd retained in the root zone [71]. Grapes take up the mineral elements they need during each growing season and heavy metals through two channels: one is through the soil root system and the other is through leaf fertilizer. Although leaves can accumulate heavy metals from atmospheric deposition and foliar fertilizers [13,72], our greenhouse experiment—without foliar application or atmospheric exposure—resulted in lower leaf Cd levels compared to field conditions.

Heavy metal uptake is influenced by both soil properties and plant factors [73], with soil type significantly affecting Cd bioavailability [74]. Stems serve as key conduits and storage organs for root-absorbed metals [33]. However, under high or prolonged Cd exposure, root metabolic function and metal uptake capacity may decline, leading to reduced root enrichment in high-concentration and longer-term treatments [75]. In fruits, the bioaccumulation factor (BCF) was higher in seeds than in pericarp and pulp, identifying seeds as the primary Cd accumulators in grape berries. Since pulp is the main consumed part and exhibits low Cd accumulation, the associated consumer risk remains relatively limited.

### 4.3. Integrated Transport and Fate of Cd in the Soil–Grape System

Heavy metals absorbed by plant roots are transported to shoots via transpiration, which generates negative pressure in the xylem and drives the upward movement of water and solutes—a key mechanism for root-to-shoot translocation of metals [76,77]. As the primary site for nutrient and Cd uptake, roots critically govern Cd mobility within the plant [78,79]. However, exogenous Cd exerts phytotoxic effects on roots, impairing their capacity to absorb and transfer both nutrients and heavy metals to aerial parts, especially with prolonged exposure [75]. Consequently, the translocation factor (TF) does not consistently rise with increasing Cd levels. In contrast, untreated grape roots—free from Cd toxicity—maintain normal nutrient and metal uptake, leading to a gradual increase in TF over time. The mobility and accumulation of Cd in plants are influenced by soil properties and plant species [80]. Soil pH, in particular, plays a decisive role: low pH increases H^+^ concentration, competing with Cd for sorption sites and enhancing its mobility, while high pH promotes the formation of Cd (OH)_2_ and reduces bioavailability [74]. Overall, alkaline conditions (pH > 8.0) favor Cd adsorption and precipitation, whereas acidic conditions (pH < 5.0) weaken Cd binding and increase its fluidity in soil [81]. Thus, soil properties fundamentally shape the mobility, bioavailability, and eventual crop accumulation of heavy metals.

### 4.4. Carcinogenic Risk (CR) of Grape Pulp and Implications for Consumer Health

To address the potential health risks associated with grape pulp consumption, we analyzed the CRI derived from grape pulp Cd accumulation across soil types, years, and population groups (Table S6; Figure 5). The USEPA defines an acceptable cancer risk threshold of 1 × 10^−6^. Our results showed that CRI of grape pulp exceeded this threshold in all scenarios, indicating non-negligible health risks: For the brown soil group in 2022, the CRI reached 1.8 × 10^−4^ (adults) and 2.5 × 10^−4^ (children)—these values are ~180-fold and 250-fold higher than the acceptable threshold, respectively, representing a significant cancer risk for consumers. Similar high CR values in soil-crop systems have been reported to correlate with elevated soil Cd bioavailability [82].

In 2023, the CRI of the Brown soil group decreased to 6.0 × 10^−5^ for adults and 8.0 × 10^−5^ for children but remained 60–80 times above the safety limit. For fluvo-aquic and red soil groups, CRI (ranging from 5.0 × 10^−6^ to 4.5 × 10^−5^) were also above the acceptable threshold, suggesting that long-term consumption of grape pulp grown in these soils may still accumulate health risks. Even low-level Cd exposure via staple or horticultural crops has been associated with chronic health hazards in human populations [83].

Notably, although grape pulp accumulates less Cd than seeds or roots, it is the primary edible part of grapes—thus, even moderate Cd accumulation in pulp translates to direct human exposure. The higher CR in adults (relative to children in some groups) may be attributed to higher daily grape consumption volumes in adult populations, consistent with dietary exposure models for fruit crops [84]. These findings highlight that grape pulp from vineyards across the three soil types carries non-negligible carcinogenic risks, especially for consumers of grapes grown in brown soil. Future management strategies (e.g., soil Cd immobilization, cultivar selection) should prioritize reducing Cd accumulation in grape pulp to mitigate consumer health risks.

## 5. Conclusions

1. Grape cultivation markedly activated soil Cd, increasing its bioavailable fractions by 1–3-fold; however, a gradual stabilization followed over time. Cd distribution within the plant exhibited a distinct pattern: root > stem > leaf > seed > skin > pulp. Although low-level Cd exposure enhanced overall accumulation, the bioaccumulation factor (BCF) in roots and stems decreased in the third year, in contrast to the increases seen in leaves, pulp, skin, and seeds. The most efficient translocation occurred to the stems, a process that was strongly suppressed under high Cd stress. Notably, in the second year, the carcinogenic risk index (CRI) of pulp from brown soil exceeded the safety threshold of 10^−4^, indicating a potential health concern.

2. Cd pollution significantly altered the soil microbial community, suppressing Actinobacteria while enriching Acidobacteria. However, with prolonged cultivation, bacterial diversity and richness increased at the genus level.

3. The influence of various environmental factors on the microbial structure diminished, and soil pH emerged as the predominant shaping force. These findings demonstrate that viticulture in contaminated soils modifies Cd speciation, plant uptake, and soil microbial ecology, with acidic soils posing a tangible consumer health risk despite grapes not being hyperaccumulators.

## 6. Limitations and Future Perspectives

Based on a three-year grape pot experiment, this study systematically analyzed the bioaccumulation characteristics of cadmium (Cd) in grapes and the corresponding human health risks. However, due to the inherent conditions of the pot experiment, the research results still have certain limitations. Firstly, there are limitations in the experimental environment. The pot experiment adopted artificially controlled soil conditions, which are significantly different from the natural field environment. Therefore, the applicability of the results in natural field scenarios needs further verification. Secondly, there are limitations in the research objects and indicators. The research conclusions on single Cd pollution are difficult to fully reflect the grape accumulation characteristics and health risks in actual pollution scenarios. Thirdly, there are limitations in the research mechanism and time scale. Although the three-year pot experiment cycle can reflect the short-to-medium-term Cd accumulation dynamics, it is difficult to cover the long-term Cd accumulation law in the entire life cycle of grapes (usually 5–10 years) and the impact of the long-term evolution of soil Cd forms on grape absorption, resulting in insufficient long-term representativeness of the research conclusions.

In view of the limitations of this study, combined with the current research hotspots in the field of soil heavy metal pollution control and agricultural product quality and safety, further research can be carried out in the following aspects in the future: Firstly, expand the experimental scenarios to improve the applicability of the research. Secondly, improve the research content and enrich the evaluation dimensions. Thirdly, conduct in-depth mechanism research to reveal the nature of accumulation. Fifthly, combine practical needs to promote technology application. This will further improve the evaluation system for Cd bioaccumulation in grapes and human health risks and provide more scientific theoretical support and technical reference for safe grape production and remediation of Cd-contaminated soils.

## Figures and Tables

**Table 1:** Basic properties of the experimental vineyard soils.

Cultivation Age	Type of Soil	Ck			Low			High		
		pH	OM g kg^−1^	T Cd mg kg^−1^	pH	OM g kg^−1^	T Cd g kg^−1^	pH	OM g kg^−1^	T Cd mg kg^−1^
2022	Fluvo-aquic soil	7.10	19.04	0.142	7.83	21.77	0.711	7.96	17.11	1.671
	Brown soil	5.59	24.10	0.100	5.52	21.02	0.367	6.08	17.41	0.661
	Red soil	7.41	7.17	0.090	8.07	9.08	0.278	7.91	4.85	0.690
2023	Fluvo-aquic soil	7.09	19.22	0.140	6.96	23.18	0.696	6.69	19.60	1.513
	Brown soil	5.98	23.92	0.091	6.40	24.59	0.366	6.11	27.99	0.656
	Red soil	6.84	10.74	0.88	6.34	11.69	0.271	7.00	10.25	0.603

**Table 2:** Concentration of exogenous Cd (mg/kg).

Type of Soil	GB15618-2018 [17]	Cd_l_	Cd_h_
Fluvo-aquic soil	0.6	0.6	1.5
Brown soil	0.3	0.3	0.75
Red soil	0.3	0.3	0.75

**Table 3:** Transfer of Cd between the root-system and the aboveground part of the grape.

Cultivation Time		CK	Low Cd	High Cd
2022	Fluvo-aquic soil	0.22291	0.3666	0.2807
	Brown soil	1.0629	2.5654	2.9295
	Red soil	0.3767	0.2815	0.1915
2023	Fluvo-aquic soil	0.4085	0.1954	0.3007
	Brown soil	1.5501	1.4483	1.2900
	Red soil	0.7031	0.8398	0.2174

## Data Availability

Data will be available on request.

## Supplementary Materials

The following supporting information can be downloaded at https://www.mdpi.com/article/10.3390/plants15071097/s1. Table S1. Soil sampling sites and soil types. Table S2. Procedure of modified BCR sequential extraction. Table S3. Microwave digester digestion process. Table S4. Alpha-diversity of bacteria at genus level in grape rhizosphere soil. Table S5. Primer sequence of 16S rRNA. Table S6. Bacterial (16S) *PCR* amplification program.
