# Supplementary material for: Bioaccumulation of Cd in Grapes and Assessment of Human Health Risk
Source: Plants (Basel). 2026 Apr 2;15(7):1097. doi: 10.3390/plants15071097 (PMC13074908; doi:10.3390/plants15071097)
Supplement: Supplementary file 1 [file plants-15-01097-s001.zip › plants-4016141-supplementary.pdf]

Title Page

Supplementary Information

Table S 1. Soil sampling sites and soil types

Soil sampling sites	Latitude and longitude	Soil type
Wu qing, Tian jin	E 116°57′28.6″ N 39°25′40.9	Fluvo-aquic soil
Ning bo, Zhe jiang	E 121°25′40.805″ N 29°57′51′13″	Brown soil
Fo shan, Guang dong	E 112°51′48.3″ N 23°31′50.9″	Red soil

Table S2. Procedure of modified BCR sequential extraction

Cd forms	Extractant	Operating conditions
Step 1 (F1)	20ml 0.1 mol/ L CH_3COOH	Room temperature oscillation 16 h
Step 2 (F2)	20ml 0.1 mol/L $NH_2OH·HCl$ (pH=2)	Room temperature oscillation 16 h
Step 3 (F3)	30% H_2O_2 and 3 ml 1mol/L CH_3COONH_4 (pH = 2)	Room temperature oscillation 1 h, 85℃<br>Water bath extraction 2 h, add NH_4Ac, oscillation 16 h
Step 4 (F4)		Total content minus sum of the fraction

Table S3. Microwave digester digestion process

heating up time	Digestion temperature	retention time
5	Room temperature-120℃	5
4	120℃-150℃	4
4	150℃-180℃	9

Table S4 Alpha-diversity of bacteria at genus level in grape rhizosphere soil

Exogenous Cd	2022		2023	
treatment	shannon	chao	shannon	chao
FCK	4.80±0.11	583.71±23.39	4.79±0.21	658.73±28.54
FL	4.70±0.11	588.49±16.28	4.73±0.16	713.74±42.36
FH	4.66±0.14	599.92±17.65	4.76±0.25	677.17±46.42
BCK	4.25±0.29	482.47±41.50	4.93±0.12	710.30±61.03
BL	4.50±0.15	501.66±37.80	4.94±0.18	687.11±28.82
BH	4.51±0.13	572.96±24.18	4.85±0.08	656.09±34.20
RCK	4.71±0.11	634.55±26.94	4.91±0.16	718.85±55.80
RL	4.66±0.13	627.68±18.67	4.74±0.14	715.55±50.41
RH	4.81±0.12	659.62±27.85	5.11±0.14	853.09±75.93

Note: FCK control group Fluvo-aquic soil; FL low Cd group Fluvo-aquic soil; FH high Cd group Fluvo-aquicl soil; BCK control group brown soil; BL low Cd group brown soil; BH high Cd group brown soil; RCK control group red soil, RL low Cd group red soil; RH high Cd group red soil;

Table S5 Primer sequence of 16S rRNA

Primer	Primer sequence
338F	ACTCCTACGGGAGGCAGCAG
806R	GGACTACHVGGGTWTCTAAT

Table S6 Bacterial (16S) *PCR* amplification program

Temperature	Hold Time	Number of Amplification Cycles
94℃	5min	28
94℃	30s	

55℃	30s
72℃	60s
72℃	7min
4℃	End
